# A comparison of machine learning techniques for survival prediction in breast cancer

**DOI:** 10.1186/1756-0381-4-12

**Published:** 2011-05-11

**Authors:** Leonardo Vanneschi, Antonella Farinaccio, Giancarlo Mauri, Mauro Antoniotti, Paolo Provero, Mario Giacobini

**Affiliations:** 1 Department of Informatics, Systems and Communication (D.I.S.Co.), University of Milano-Bicocca, Milan, Italy; 2 Computational Biology Unit, Molecular Biotechnology Center, University of Torino, Italy; 3 Department of Genetics, Biology and Biochemistry, University of Torino, Italy; 4 Department of Animal Production, Epidemiology and Ecology, University of Torino, Italy

## Abstract

**Background:**

The ability to accurately classify cancer patients into risk classes, i.e. to predict the outcome of the pathology on an individual basis, is a key ingredient in making therapeutic decisions. In recent years gene expression data have been successfully used to complement the clinical and histological criteria traditionally used in such prediction. Many "gene expression signatures" have been developed, i.e. sets of genes whose expression values in a tumor can be used to predict the outcome of the pathology. Here we investigate the use of several machine learning techniques to classify breast cancer patients using one of such signatures, the well established *70-gene signature*.

**Results:**

We show that Genetic Programming performs significantly better than Support Vector Machines, Multilayered Perceptrons and Random Forests in classifying patients from the NKI breast cancer dataset, and comparably to the scoring-based method originally proposed by the authors of the 70-gene signature. Furthermore, Genetic Programming is able to perform an automatic feature selection.

**Conclusions:**

Since the performance of Genetic Programming is likely to be improvable compared to the out-of-the-box approach used here, and given the biological insight potentially provided by the Genetic Programming solutions, we conclude that Genetic Programming methods are worth further investigation as a tool for cancer patient classification based on gene expression data.

## Background

Current cancer therapies have serious side effects: ideally type and dosage of the therapy should be matched to each individual patient based on his/her risk of relapse. Therefore the classification of cancer patients into risk classes is a very active field of research, with direct clinical applications. Until recently patient classification was based on a series of clinical and histological parameters. The advent of high-throughput techniques to measure gene expression led in the last decade to a large body of research on gene expression in cancer, and in particular on the possibility of using gene expression data to improve patient classification. A gene signature is a set of genes whose levels of expression can be used to predict a biological state (see [1]): in the case of cancer, gene signatures have been developed both to distinguish cancerous from non-cancerous conditions and to classify cancer patients based on the aggressiveness of the tumor, as measured for example by the probability of relapsing within a given time.

While many studies have been devoted to the identification of gene signatures in various types of cancer, the question of the algorithms to be used to maximize the predictive power of a gene signature has received less attention. To investigate this issue systematically, we considered one of the best established gene signatures, the 70-gene signature for breast cancer [2], and we compared the performance of four different machine learning algorithms in using this signature to predict the survival of a cohort of breast cancer patients. The 70-gene signature is a set of microarray features selected in [2] based on correlation with survival, on which the molecular prognostic test for breast cancer "MammaPrint"™ is based. While several machine learning algorithms have been used to classify cancer samples based on gene expression data [3-8], in this work we performed a systematic comparison of the performance of four machine learning algorithms using the same features to predict the same classes. In our comparison, feature selection is thus *not *explicitly performed as a pre-processing phase before executing the machine learning algorithms^1^. We considered GP, Support Vector Machines, Multilayered Perceptrons and Random Forests, and we applied them to the problem of using the 70-gene signature to predict the survival of the breast cancer patients included in the NKI dataset [9]. This is considered one of the gold-standard datasets in the field, and the predictive power of the 70-gene signature on these patients was already shown in [9]. In this preliminary study we tried to use all the methods in an "out-of-the-box" version so as to obtain a first evaluation, as unbiased as possible, of the performance of the methods.

## Previous and Related Work

Many different machine learning methods [10] have already been applied for microarray data analysis, like k-nearest neighbors [11], hierarchical clustering [12], self-organizing maps [13], Support Vector Machines [14,15] or Bayesian networks [16]. Furthermore, in the last few years Evolutionary Algorithms (EA) [17] have been used for solving both problems of feature selection and classification in gene expression data analysis. Genetic Algorithms (GAs) [18] have been employed for building selectors where each allele of the representation corresponds to one gene and its state denotes whether the gene is selected or not [19]. GP on the other hand has been shown to work well for recognition of structures in large data sets [20]. GP was applied to microarray data to generate programs that reliably predict the health/malignancy states of tissue, or classify different types of tissues. An intrinsic advantage of GP is that it automatically selects a small number of feature genes during the evolution [21]. The evolution of classifiers from the initial population seamlessly integrates the process of gene selection and classifier construction. In fact, in [8] GP was used on cancer expression profiling data to select potentially informative feature genes, build molecular classifiers by mathematical integration of these genes and classify tumour samples. Furthermore, GP has been shown a promising approach for discovering comprehensible rule-based classifiers from medical data [22] as well as gene expression profiling data [23]. The results presented in those contributions are encouraging and pave the way to a further investigation of GP for this kind of datasets, which is the goal of this paper.

## Results and Discussion

### Predictive Power of Machine Learning Methods

We used the NKI breast cancer dataset [9], providing gene expression and survival data for 295 consecutive breast carcinoma patients. We considered only the expression data for the genes included in the "70-gene" signature [2].

Both survival and gene expression data were transformed into binary form. For the survival data, we defined the outcome as the survival status of the patient at time *t_end _*= 10.3 years. By choosing this particular endpoint we balanced the number of dead and alive patients: out of 148 patients for which the status at *t_end _*is known, 74 were dead and 74 were alive. Binary expression data were obtained by replacing all positive logarithmic fold changes in the original dataset with 1 and all negative and missing ones with 0.

Our dataset is a matrix *H *= [*H*_(*i*, *j*)_] of binary values composed by 148 rows (instances) and 71 columns (features), where each line *i *represents the gene signature of a patient whose binary target (0 = survived after *t_end _*years, 1 = dead for breast cancer before *t_end _*years) has been placed at position *H*_(*i*,71)_. In this way, the last column of matrix *H *represents all the known target values. Our task is now to generate a mapping *F *such that *F *(*H*_(*i*,1)_, *H*_(*i*,2) _, ..., *H *_(i,70)_) = *H*_(*i*,71) _for each line *i *in the dataset. Of course, we also want *F *to have a good generalization ability, i.e. to be able to assess the target value for new patients, that have not been used in the training phase. For this reason, we used a set of machine learning techniques, as discussed in Section Methods. To compare the predictive power of the computational methods, we performed 50 independent choices of training and test set, the training set including 70% of the patients and the test set the remaining 30%. The various prediction methods were then run on these datasets, so that the choice of training and testing sets in each run was the same for all methods.

Table 1 summarizes the results returned by each machine learning method on the 50 runs. The first line indicates the different methods, the second line shows the best (i.e. lowest) value of the incorrectly classified instances obtained on the test set over the 50 runs, and the third line reports the mean performances of each group of 50 runs on their test sets, together with the corresponding standard error of mean (SEM).

**Table 1:** Experimental comparison between the number of incorrectly classified instances found on the test sets by the different machine learning methods.

	GP	SVM-K1	SVM-K2	SVM-K3	MP	RF
best	10	13	14	15	10	12

average (SEM)	16.40 (0.30)	18.32 (0.37)	16.76 (0.18)	17.62 (0.17)	18.08 (0.39)	17.60 (0.35)

Each method was independently run 50 times using each time a different training/test partition of the validation dataset (see text for details). The first line indicates the method: Genetic Programming (GP), Support Vector Machine with exponent for the polynomial kernel 1.0 (SVM-K1), 2.0 (SVM-K2), and 3.0 (SVM-K3), Multilayer Perceptrons (MP), and Random Forest (RF). The second line shows the best value of the incorrectly classified instances obtained on the test set over the 50 runs, and the third line reports the average performances of each group of 50 runs on their test sets (standard error of mean is shown in parentheses).

As Table 1 clearly shows, the best solutions were found by GP and Multilayered Perceptrons and the best average result was found by GP. Moreover, statistical analysis indicates that GP consistently outperforms the other methods except SVM using polynomial kernel with degree 2. In fact, as it can be seen in Table 2, the difference between the various average results is statistically significant (P-value 3.05 × 10^-5 ^for ANOVA test on the 4 samples of solutions found by each method). Finally, pairwise 2-tailed Student t-tests comparing GP with each other method demonstrate its general better performance. These statistical tests were performed since there was no evidence of deviation from normality or unequal variances.

**Table 2:** Statistical significance of the difference in performance between the methods.

ANOVA *P *= 3.05 × 10^-5^				
GP vs. SVM-K1 *P *= 0.0001	GP vs. SVM-K2 *P *= 0.3107	GP vs. SVM-K3 *P *= 0.0008	GP vs. MP *P *= 0.0009	GP vs. RF *P *= 0.0103

First line shows ANOVA test on the 6 samples of solutions found by each method, while second line depicts pairwise 2-tailed Student t-tests comparing GP with each other method.

The solutions found by GP typically use a rather small number of features (i.e. terminals). In fact, the solutions of the 50 GP runs are functions of a number of terminal that ranges from 1 to 23, with a median value of 4, and first and third quartiles of 2 and 7 respectively. Few of these features tend to recur in several solution as it can be seen in Table 3, where the gene symbol, the gene name of each feature, together with the number of solutions where the feature occurs are shown.

**Table 3:** The 10 most recurring features in the solutions found by GP.

Accession ID	Gene name	Gene description	Solutions
NM_003981	PRC1	protein regulator of cytokinesis 1	48

NM_002916	RFC4	replication factor C (activator 1) 4, 37 kDa	23

AI992158	-	-	16

AI554061	-	-	10

NM_006101	NDC80	NDC80 homolog, kinetochore complex component (S. cerevisiae)	9

NM_015984	UCHL5	ubiquitin carboxyl-terminal hydrolase L5	7

NM_020188	C16orf61	chromosome 16 open reading frame 61	6

NM_016448	DTL	denticleless homolog (Drosophila)	6

NM_014791	MELK	maternal embryonic leucine zipper kinase	6

NM_004702	-	-	6

The four columns show: accession ID, gene name, gene description, and number of solutions where that feature occurs.

### Comparison with the Scoring Method

The authors of Ref. [9] used the seventy-gene signature by computing the correlation coefficient between the expression profile of the patient (limited to the 70 genes of the signature) and a previously computed typical expression profile of a good prognosis patient. To compare the performance of the various machine learning algorithms with this scoring system we proceeded as follows:

• We obtained the prognostic score s of the patients (excluding the ones used to train the signature in [2]) from the Supplementary Material of [9], and classified as good prognosis the patients with *s *> 0.4 and as bad prognosis the ones with *s *≤ 0.4. This is the cutoff used in [9].

• We generated 50 random lists of 44 patients from this set, to match the statistic used for machine learning techniques, and computed for each list the number of false predictions given by the scoring method.

The mean number of false predictions was 16.24, with a SEM of 0.37. Therefore the scoring method appears to be superior to all machine learning algorithm other than GP, and slightly superior to GP. The difference between the performances of GP and the scoring method are not statistically significant (*P *= 0.49, 2-tailed Student t-test).

### The Role of Feature Selection

To determine to what extent feature selection is responsible for the good performance of GP, we identified the 10 features most often selected by GP among the 70 initial features and ran again both GP and SVM with quadratic kernel using only these features. The performance of *both *methods significantly improved: for GP, the number of incorrectly identified features decreased from 16.40 (SEM 0.30) to 12.86 (0.40); for the SVM it went from 16.76 (0.18) to 14.96 (0.41). Using this preliminary round of feature selection the performance of GP becomes significantly better than both SVM and the original scoring method.

These results suggests on one hand, that the feature selection performed by GP has intrinsic value, not necessarily tied to the use of syntax trees, since the SVM can take advantage of the feature selection performed by GP to improve its performance. Second, that a recursive use of GP, in which a first run is used to select the best features to be used in a second run, might be a promising way of optimizing the method.

### Performance on Unbalanced Datasets

To check whether the performance of the GP is tied to the choice of a balanced dataset, we repeated the analysis using different time cutoffs (5 and 7.5 years) and compared the performance of GP with the SVM using polynomial kernel with degree 2, which was the best performing method after GP in the balanced dataset. The results are reported in Table 4. At 7.5 years there is again no significant difference between the performance of the two methods. However, at 5 years GP performs significantly better than the SVM (*P *= 6.46 × 10^-6 ^from two-sided *t*-test). We conclude that the balancing of the dataset is not crucial to obtain a good performance from GP.

**Table 4:** Experimental comparison between the number of incorrectly classified instances found on the test sets by GP and Support Vector Machine with exponent 2 on unbalanced datasets.

	5 yrs		7.5 yrs	
	**GP**	**SVM-K2**	**GP**	**SVM-K2**

best	9	10	12	13

average (SEM)	15.04 (0.41)	17.84 (0.42)	21.18 (0.49)	20.7 (0.46)

The datasets are defined by survival status at endpoints 5 and 7.5 years.

### Performance on an Independent Dataset

An important feature of any predictor based on gene expression data is its robustness with respect to the choice of dataset, since gene expression data from cancer patients come from studies using different protocols and/or microarray platforms. We thus applied the best predictors found by GP in each of the 50 runs to an independent breast cancer dataset [24]. It includes 251 breast cancer samples hybridized onto the Affymetrix HG-U133A and HG-U133B plat-forms. Gene expression and clinical data are publicly available in the Gene Expression Omnibus archive [25] under accession GSE3494. Due to the difference in gene content between platforms, only 17 of the 50 best solutions found by GP could be applied to the new dataset. We then constructed, for each GP solution, a 2 × 2 contingency table comparing the GP prediction to the true outcome at 10 years and applied the exact Fisher test to the table. All 17 GP solutions showed statistically significant predictive power (P-values between 7.6 × 10^-3 ^and 2.9 × 10^-4^). Since this result was obtained with no further training, it shows the robustness of the solutions obtained by GP with respect to the choice of dataset and microarray platform.

### Assessment of Sensitivity

When using gene signatures to predict the survival of a cohort of breast cancer patients, one of the main goal in clinical applications is to minimize the number of false negative predictions. Table 5 summarizes the false negative predictions returned by each machine learning method on the 50 runs. The first line indicates the different methods, while the second and the third lines show the best (i.e. lowest) and mean performances (together with the corresponding SEM)values of incorrectly classified instances.

**Table 5:** Experimental comparison between the number of false negatives found on the test sets by the different machine learning methods.

	GP	SVM-K1	SVM-K2	SVM-K3	MP	RF
best	2	6	6	6	5	6

average (SEM)	9.82 (0.44)	13.26 (0.51)	12.60 (0.35)	14.08 (0.39)	12.88 (0.51)	13.38 (0.49)

Each method was independently run 50 times using each time a different training/test partition of the validation dataset (see text for details). The first line indicates the method: Genetic Programming (GP), Support Vector Machine (SVM), Multilayer Perceptrons (MP), and Random Forest (RF). The second line shows the best value of the incorrectly classified instances obtained on the test set over the 50 runs, and the third line reports the average performances of each group of 50 runs on their test sets (standard error of mean is shown in parentheses).

The best solutions were found by GP, and statistical analysis indicates that GP consistently outperforms the other five methods as it can be seen in Table 6. The difference between the various average results is statistically significant (P-value 2.75 × 10^-9 ^for ANOVA test on the 4 samples of solutions found by each method). Finally, pairwise 2-tailed Student t-tests comparing GP with each other method demonstrate its better performance.

**Table 6:** False negative prediction: statistical significance of the difference in performance between the methods.

ANOVA *P *= 2.75 × 10^-9^				
GP vs. SVM-K1	GP vs. SVM-K2	GP vs. SVM-K3	GP vs. MP	GP vs. RF
*P *= 2.74 × 10^-6^	*P *= 3.32 × 10^-6^	*P *= 1.27 × 10^-10^	P = 8.53 × 10^-6^	*P *= 4.65 × 10^-7^

First line shows ANOVA test on the 6 samples of solutions found by each method, while second line depicts pairwise 2-tailed Student t-tests comparing GP with each other method.

The original scoring method of [2,9], and in particular the suggested cutoff of 0.4, was chosen in such a way as to minimize the number of false negatives. Therefore it is not surprising that in this respect the scoring method is far superior to all machine learning methods, including GP. Indeed the average number of false negatives given by the scoring method is 1.78, to be compared to the numbers reported in Table 6.

### Maximizing Sensitivity in GP

It is well know that the fitness function driving the evolutionary dynamics in a GP framework can be modified in order to let emerge solutions with different characteristics. The results presented and discussed in the previous section were obtained with the goal of minimizing all incorrectly classified instances, summing both false negative and false positive predictions obtained by the solutions. However, when using gene signatures to predict the survival of a cohort of breast cancer patients, minimizing the number of false negative predictions is recognized as one of the most important goals.

For all these reasons, we modified the GP fitness function so that false negatives (positives) are penalized more than errors of the other type, hoping to tune the algorithm towards better sensitivity (sensibility). In particular, solutions with greater sensitivity can emerge if larger weights are assigned to false negatives compared to false positives. In general, we can transform the fitness function in a weighted average of the form:

With respect to this new formulation, the fitness function of the GP algorithm whose results were presented in the previous section can be expressed as 0.9 × *FalseNegative *+ 0.1 × *FalsePositive*. The results of 50 runs of this new version of the GP technique showed an average of 16.04 (with SEM = 0.44) of total incorrectly classified instances. Compared with the performances of the previous GP algorithm, no statistically significant difference can be highlighted (Student t-test *P *= 0.50). When looking only at the number of false negative incorrectly classified instances, the average performance of 4.32 (SEM = 0.346) is better than the one of standard GP reported in table 6 (Student t-test *P *= 6.62 × 10^-16^), even if still worse than that of the original scoring method.

## Conclusions

The goal of our investigation was to refine the set of criteria that could lead to better risk stratification in breast cancer. To reach this goal we started from the well known "70-genes signature" and proceeded with the application of several machine learning schemes, in order to perform a comparison between them. We made some simplifying assumptions, preprocessed the data accordingly and ran several evaluation experiments.

Our results showed that while all the machine learning algorithms we used do have predictive power in classifying breast cancer patients into risk classes, GP clearly outperforms all other methods with the exception of SVM with polynomial kernel of degree 2, whose performance is not significantly different from GP. Of course there is no way to do such a comparison in a completely unbiased way, as one could always argue that the levels of optimization are uneven. To minimize the possible bias, we tried to use default implementation of all the methods.

The survival endpoint was initially chosen so as to produce a balanced dataset with the same number of samples in each outcome class. However this choice turned out not to be crucial to the good performance of GP since also on unbalanced datasets GP turned out to perform comparably to or better than the SVM. Moreover, the predictive solutions found by GP in a dataset turned out to be significantly predictive of survival also in another, independent dataset without any further training.

A unique characteristic of GP is its ability to perform automatic feature selection. We showed that the feature selection performed by GP was quite dramatic (the median number of features used by the best GP solution was ~4 out of a total of 70 features available) and of intrinsic value, *i.e*. not necessarily tied to the use of syntax trees: indeed the performance of both GP and SVM significantly improved when run using only the features selected most often in a preliminary GP run.

The improvement in performance shown by GP compared to the original scoring method was rather small and not statistically significant. As expected, the scoring method was superior to all machine learning algorithms in minimizing false negatives. In a second phase, we tried to enrich GP by changing its fitness function into a weighted average between false negatives and false positives. We showed that, when larger weight is given to false negatives, it is possible to tune the GP algorithm towards greater sensitivity. While the sensitivity of GP is still less than the original scoring method, the possibility of tuning the fitness function is another intrinsic advantage of this technique with respect to the other machine learning ones considered in this article.

**Figure 1:**
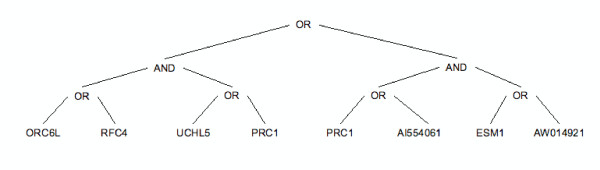
**The best-fitness model**. Tree representation and the traditional Lisp representation of the model with the best fitness found by GP over the studied 50 independent runs.

Nevertheless we believe our results warrant further investigation into the use of GP in this context for at least three reasons:

• As stated above, our implementation of GP was purposely not optimized, and we can expect substantial improvements in performance from further work aimed at tuning the various GP parameters.

• Maybe more importantly, GP can potentially offer biological insight and generate hypotheses for experimental work (see also [8]). Indeed an important result of our analysis is that the trees produced by GP tend to contain a limited number of features, and therefore are easily interpretable in biological terms. For example, the bestperforming tree is shown in Figure 1 and includes 7 genes (features).

• Finally within the context of GP there is a natural way to tune the algorithm towards better sensitivity (specificity), simply by defining a fitness function in which false negatives (positives) are penalized more than errors of the other type.

Future work along these lines should therefore focus on both improving the performance of GP and interpreting the results from the biological point of view. An obvious first step towards optimization would be to abandon the binarization of the data (which here was used to produce trees that are easier to interpret) and build a GP based on continuous expression values. The biological interpretation might benefit from a statistical and functional analysis of the most recurring subtrees in optimal GP solutions.

In conclusion we have shown that Genetic Programming outperforms other machine learning methods as a tool to extract predictions from an established breast cancer gene signature. Given the possibility of generating biological insight and hypotheses that is intrinsic to the method, it deserves deeper investigation along the lines described above. Finally, it will be our task to test the GP approach on other features/gene sets that account for other cancers or other diseases, always with the objective of providing clinicians with more precise and individualized diagnosis criteria.

## Methods

The machine learning methods we considered are described here, with references to more detailed expositions.

### Genetic Programming

Genetic Programming (GP) [26-28] is an evolutionary approach which extends Genetic Algorithms (GAs) [17,18] to the space of programs. Like any other evolutionary algorithm, GP works by defining a goal in the form of a quality criterion (*or fitness*) and then using this criterion to *evolve *a set (also called population) of solution candidates (also called individuals) by mimic the basic principles of Darwin's theory of evolution [29]. The most common version of GP, and also the one used here, considers individuals as *abstract syntax tree *structures^2 ^that can be built recursively from a set of function symbols ℱ = {*f*_1_, *f*_2_, ..., *f_n_*} (used to label internal tree nodes) and a set of terminal symbols  (used to label tree leaves). GP breeds these solutions to solve problems by executing an iterative process involving the probabilistic selection of the fittest solutions and their variation by means of a set of genetic operators, usually crossover and mutation.

We used a tree-based GP configuration inspired by boolean problems introduced in [26]: each feature in the dataset was represented as a boolean value and thus our set of terminals  was composed by 70 boolean variables (i.e. one for each feature of our dataset). Potential solutions (GP individuals) were built using the set of boolean functions ℱ = {*AND*, *OR*, *NOT*}. The fitness function is the number of incorrectly classified instances, which turns the problem into a minimization one (lower values are better)^3^.

Finally no explicit feature selection strategy was employed, since we want to point out GP's ability to automatically perform an implicit feature selection. The mechanism allowing GP to perform feature selection, already pointed out for instance in [21,30-32], is simple: GP searches over the space of all boolean expressions of 70 variables. This search space includes the expressions that use *all *the 70 variables, but also the ones that use a *smaller *number of variables. In principle there is no reason why an expression using a smaller number of variables could not have a better fitness value than an expression using all the 70 variables. If expressions using smaller number of variables have a better fitness, they survive into the population, given that fitness is the only principle used by GP for selecting genes. If it happens that GP finds expressions using a small number of variables with a better fitness value than the ones using all variables, the former expressions survive into the population, while the latter ones are extinguished.

The parameters used in our GP experiments are reported in Table 7, together with the parameters used by the other machine learning methods we studied. There is no particular justification for the choice of those parameter values, if not the fact that they are standard for the computational tool we used, i.e. GPLab: a public domain GP system implemented in MatLab (for the GPLab software and documentation, see [33]).

**Table 7:** Parameters used in the experiments.

GP Parameters	
population size	500 individuals

population initialization	ramped half and half [26]

selection method	tournament (tournament size = 10)

crossover rate	0.9

mutation rate	0.1

maximum number of generations	5

algorithm	generational tree based GP with no elitism

**SVM Parameters**	

complexity parameter	0.1

size of the kernel cache	10^7^

epsilon value for the round-off error	10^-12^

exponent for the polynomial kernel	1.0,2.0, 3.0

tolerance parameter	0.001

**Multilayered Perceptron Parameters**	

learning algorithm	Back-propagation

learning rate	0:03

activation function for all the neurons in the net	sigmoid

momentum	0.2 progressively decreasing until 0.0001

hidden layers	(number of attributes + number of classes)/2

number of epochs of training	500

**Random Forest Parameters**	

number of trees	2500

number of attributes per node	1

### Support Vector Machines

Support Vector Machines (SVM) are a set of related supervised learning methods used for classification and regression. They were originally introduced in [34]. Their aim is to devise a computationally efficient way of identifying separating hyperplanes in a high dimensional feature space. In particular, the method seeks separating hyperplanes maximizing the margin between sets of data. This should ensure a good generalization ability of the method, under the hypothesis of consistent target function between training and testing data. To calculate the margin between data belonging to two different classes, two parallel hyperplanes are constructed, one on each side of the separating hyperplane, which are "pushed up against" the two data sets. Intuitively, a good separation is achieved by the hyperplane that has the largest distance to the neighboring data points of both classes, since in general the larger the margin the lower the generalization error of the classifier. The parameters of the maximum-margin hyperplane are derived by solving large quadratic programming (QP) optimization problems. There exist several specialized algorithms for quickly solving these problems that arise from SVMs, mostly reliant on heuristics for breaking the problem down into smaller, more manageable chunks. In this work we used the implementation of John Platt's [35] sequential minimal optimization (SMO) algorithm for training the support vector classifier. SMO works by breaking the large QP problem into a series of smaller 2-dimensional sub-problems that may be solved analytically, eliminating the need for numerical optimization algorithms such as conjugate gradient methods. The implementation we used is the one contained in the Weka public domain software [36]. This implementation globally replaces all missing values and transforms nominal attributes into binary ones. It also normalizes all attributes by default (in that case the coefficients in the output are based on the normalized data, not the original data and this is important for interpreting the classifier).

The main parameter values used in this work are reported in Table 7. All these parameter values correspond to the standard values offered by the Weka software [36] and they are defined for instance in [35]. Being aware that in several application domains, SVM have been shown to outperform competing techniques by using nonlinear kernels, which implicitly map the instances to very high (even infinite) dimensional spaces, we used polynomials kernels with degrees 1, 2, and 3.

### Multilayered Perceptron

Multilayered Perceptron is a feed-forward artificial neural network model [37]. It is a modification of the standard linear perceptron in that it uses three or more layers of neurons (nodes) with nonlinear activation functions, and is more powerful than simple perceptron in that it can distinguish data that are not linearly separable, or separable by a hyperplane. It consists of an input and an output layer with one or more hidden layers of nonlinearly-activating nodes. Each node in one layer connects with a certain weight to every other node in the following layer. The implementation we have adopted is the one included in the Weka software distribution [36]. We used the Back-propagation learning algorithm [37] and the values used for all the parameters are reported in Table 7. As for the previously discussed machine learning methods, also in the case of Multilayered Perceptron it is important to point out that we used a parameter setting as standard as possible, without doing any fine parameter tuning for this particular application. Our goal is, in fact, to compare different computational methods under standard conditions and not to solve in the best possible way the application itself. In particular, all the values reported in Table 7 correspond to the default ones adopted by the Weka software.

### Random Forests

Random Forests denotes an improved Classification and Regression Trees method [38]. It works by creating a large number of classification trees or regression trees. Every tree is built using a deterministic algorithm and the trees are different owing to two factors. First, at each node, a best split is chosen from a random subset of the predictors rather than from all of them. Secondly, every tree is built using a bootstrap sample of the observations. The out-of-bag data, approximately one-third of the observations, are then used to estimate the prediction accuracy. Unlike other tree algorithms, no pruning or trimming of the fully grown tree is involved. In this work we use the Breiman model presented in [39] and implemented in the Weka software [36]. As it can be seen from Table 7, this method, compared to the other ones, has the advantage of a smaller amount of parameter setting required. In order to allow a fair comparison with GP, we have considered random forests composed by 2500 trees (given that the GP population is composed by 500 trees and it runs for 5 generations, 2500 trees are globally inspected by GP too) and such that each node corresponds to exactly one feature (as it is for GP). All the other parameters eported in Table 7 were set to the standard values offered by the Weka software.

## Competing interests

The authors declare that they have no competing interests.

## Authors' contributions

LV, PP, and MG conceived the design of the study, participated in the interpretation of the results, and coordinated the participants' contributions. AF participated in the design of the study, implemented the computational model and carried out the simulations. GM and MA participated in the design of the study. All authors equally participated in writing the manuscript and approved it.

## Endnotes

1. As we will discuss later, Genetic Programming (GP) is the only method, among the ones studied in this paper, hat is able to perform automatically a further feature selection and thus identify small subsets of the original signature characterized by high predictive power.

2. Traditionally represented in Lisp notation.

3. We are aware that, in case of minimization problems, the term "fitness" might be inappropriate, given that a fitness is usually a measure that has to be maximized. Nevertheless, we chose to use this term for simplicity.
